# Chitins from Seafood Waste as Sustainable Porous Carbon Precursors for the Development of Eco-Friendly Supercapacitors

**DOI:** 10.3390/ma16062332

**Published:** 2023-03-14

**Authors:** Ana T. S. C. Brandão, Renata Costa, Sabrina State, Pavel Potorac, Catarina Dias, José A. Vázquez, Jesus Valcarcel, A. Fernando Silva, Marius Enachescu, Carlos M. Pereira

**Affiliations:** 1 Instituto de Ciências Moleculares IMS-CIQUP, Departamento de Química e Bioquímica, Faculdade de Ciências da Universidade do Porto, Rua do Campo Alegre, 687, 4169-007 Porto, Portugal; 2 Center for Surface Science and Nanotechnology, University Polytechnica of Bucharest, Splaiul Independentei, 313, 060042 Bucharest, Romania; 3 Grupo de Reciclado y Valorización de Residuos (REVAL), Instituto de Investigaciones Marinas (IIM-CSIC), 36208 Vigo, Spain; 4 Academy of Romanian Scientists, Splaiul Independentei 54, 050094 Bucharest, Romania

**Keywords:** marine biomass, chitins, biocarbon, carbonization process, deep eutectic solvents, specific capacitance

## Abstract

Carbon materials derived from marine waste have been drawing attention for supercapacitor applications. In this work, chitins from squid and prawn marine wastes were used as carbon precursors for further application as electrodes for energy storage devices. Chitins were obtained through a deproteinization method based on enzymatic hydrolysis as an alternative to chemical hydrolysis as commonly presented in the literature. The obtained porous carbons were characterized using a BET surface area analyzer to determine the specific surface area and pore size, as well as scanning electron microscopy (SEM) with energy dispersive X-ray analysis (EDX), transmission electron microscopy (TEM), Raman spectroscopy, attenuated total reflectance Fourier transform infrared (ATR-FTIR) spectroscopy, X-ray diffraction (XRD), and X-ray photoelectron spectroscopy (XPS), to characterize their morphology, composition, and structure. The electrochemical characterization was performed using a glassy carbon (GC) electrode modified with marine waste-based porous carbons as the working electrode through cyclic voltammetry and galvanostatic charge/discharge using ethaline, a choline chloride-based deep eutectic solvent (DES), as an eco-friendly and sustainable electrolyte. Squid and prawn chitin-based carbons presented a surface area of 149.3 m^2^ g^−1^ and 85.0 m^2^ g^−1^, pore volume of 0.053 cm^3^ g^−1^ and 0.029 cm^3^ g^−1^, and an associated specific capacitance of 20 and 15 F g^−1^ at 1 A g^−1^, respectively. Preliminary studies were performed to understand the effect of -OH groups on the chitin-based carbon surface with DES as an electrolyte, as well as the effect of aqueous electrolytes (1 mol L^−1^ sulphuric acid (H_2_SO_4_) and 1 mol L^−1^ potassium hydroxide (KOH)) on the capacitance and retention of the half-cell set up. It is provided, for the first time, the use of chitin-based carbon materials obtained through a one-step carbonization process combined with an eco-friendly DES electrolyte for potential application in energy storage devices.

## 1. Introduction

The energy and climate crisis alongside the increase in energy consumption and understanding of environmental challenges have enforced the demand for sustainable development of clean and high-performance materials for energy storage applications [1]. Among the different energy storage device configurations available, supercapacitors are energy storage devices with outstanding properties, such as fast charge/discharge rates, superior power density, and extended cycle life [2]. The electrode materials and the electrolyte play an essential role in the energy storage system performance of the supercapacitors. Carbon materials are preferentially used as electrodes in supercapacitors due to their excellent conductivity, chemical stability, high surface area, and porosity [3]. To avoid the continuous deterioration and exploitation of environmental resources that bring a negative impact on the ecosystem and local communities, the innovation towards more eco-friendly sources and protocols to produce carbon materials has been gaining more attention in the last few years [1,3,4,5]. Among them, carbons based on biomass waste are described as renewable resources with unique structures [6]. Animal waste materials can act as carbon precursors to generate renewable energy while at the same time preventing the leaching of hazardous wastes into the environment. Chitin is mainly found in marine biomass waste, such as the shells of crabs or shrimp, and presents adequate nitrogen content of ~6.9 wt.% [7] compared to ~2 wt.% when it comes from prawns and squid [8]. Normally, marine waste-derived materials are rich in heteroatoms, such as nitrogen and oxygen.

The synthesis and applications of carbon materials prepared from biomass and marine waste biomass are widely reviewed [9,10,11,12,13,14,15,16,17]. Several studies focused on the performance of porous carbon-based electrodes from fish waste for supercapacitor applications have been presented, applying different types of fish waste sources, ranging from crab shells, fish and prawn scales, fish bones, and others [18,19,20,21,22,23,24]. Some publications exploring the handling of chitin-based carbons for supercapacitor practical applications can be found in the literature [25,26,27], exhibiting promising results for upscaling and large-scale applications. Raj et al. [19] synthesized oxygen and nitrogen-doped activated carbons using chitin from the gladius of squid fish, creating an activated carbon with a high surface area of 1129 m^2^ g^−1^ with a maximum specific capacitance of 204 F g^−1^ in 1 M H_2_SO_4_ electrolyte. Wang et al. [28] synthesized heteroatoms-doped hierarchical porous carbons derived from chitin using KMnO_4_ as activating agent, achieving a maximum surface area of 1488.3 m^2^ g^−1^ with a specific capacitance of 412.5 F g^−1^ at 0.5 A g^−1^ (0.4% loss after 10,000 cycles). Concerning the application of these materials to supercapacitors, it is also essential to highlight the importance of the electrolyte.

Deep eutectic solvents (DESs) present several advantages when compared to aqueous electrolytes, including better electrochemical stability, wider potential windows, less impact on the ecosystem, etc., leading to this innovative class of nonaqueous/dense ionic fluids becoming the electrolytes of choice for supercapacitors [29,30,31,32,33,34,35,36,37,38].

In this work, a correlation is established between the chitin source (using prawns and squids as the starting materials) and the electrochemical performance of the system. Accordingly, biocarbons were prepared from the chitin extracted from prawns and squids by a one-step carbonization process with the time and temperature strictly controlled (1 h at 1000 °C). The resulting biocarbon materials were characterized using a BET surface area analyzer, scanning electron microscopy (SEM) with energy dispersive X-ray analysis (EDX), transmission electron microscopy (TEM), Raman spectroscopy, attenuated total reflectance Fourier transform infrared (ATR-FTIR) spectroscopy, X-ray diffraction (XRD), and X-ray photoelectron spectroscopy (XPS). The electrochemical analysis was performed using cyclic voltammetry and galvanostatic charge/discharge chitin composite electrodes/choline chloride-based DES electrolyte (ethylene glycol as hydrogen bond donor). Simultaneously, two aqueous electrolytes (sulphuric acid (H_2_SO_4_) and potassium hydroxide (KOH)) were used to make a proper comparison of the performance between aqueous- and the deep-eutectic-based electrolytes. The activation of the squid-derived biocarbons was also assessed through the introduction of OH- groups using NaOH as an activating agent. These materials derived from chitin and associated with an eco-friendly electrolyte, may be a step forward to the development of safer and more reliable energy storage devices that are efficient in charge and discharge with a longer cycle life, bringing many benefits to the world’s storage/conversion of clean energy based on the fundamental pillars circular economy.

## 2. Materials and Methods

### 2.1. Biocarbon Derived from Fish Waste (Prawn and Squid Chitins) Preparation

Cephalothorax of King prawn (*Penaeus vannamei*) and (*Illex argentinus*) were obtained as byproducts from the industrial processing of both species and kindly provided by Pescanova S.A. (Vigo, Spain) and Cabomar S.A. (Marín, Spain). In the case of shells of prawn, the production of chitin was performed according to Vázquez et al. [39]. Briefly, shells were ground (0.5–1.5 cm), washed to eliminate the rest of the crustacean body, deproteinized by Alcalase^®^ 2.4 L FG (Novozymes, Novodirsk, Bagsværd, Denmark) hydrolysis, demineralized with HCl twice, purified with NaOH treatment, neutralized with an intense water wash, depigmented using NaClO wash, and the α-chitin obtained was finally dried in an oven. By contrast, pens were ground and sieved (0.5 cm), deproteinized with Alcalase, and the recovered β-chitin was also dried in an oven [40]. 

The raw biomass was placed inside a tubular furnace at 1000 °C for 1 h with a 0.3 L h^−1^ N_2_ flow-controlled environment to further collect the ashes (39 wt.% recoveries). The variation of time and temperature of carbonization was applied, and preliminary results were obtained. The temperature and time of 1000 °C and 1 h presented the best results, so these parameters were chosen to proceed with this study. The results are presented in Table S1 in Supporting Information.

### 2.2. Morphological Characterization of the Carbon Materials

The morphological characterization of the chitin-based carbon materials was assessed through scanning electron microscopy (SEM) with energy dispersive X-ray analysis (EDX) (Hitachi SU 8230 equipment, Krefeld, Germany), transmission electron microscopy (TEM, Hitachi H-8100 equipment, Tokyo, Japan), Raman spectroscopy (Ramos PA532 Ostec, Moscow, Russia), attenuated total reflectance Fourier transform infrared spectroscopy (ATR-FTIR, Systems Chemistry, Heyendaalseweg, Netherlands), X-ray diffraction (XRD, Rigaku SmartLab X-ray Diffractometer, Rigaku Corporation, Tokyo, Japan), X-ray photoelectron spectroscopy (XPS, Kratos Analytical Ltd., Manchester, UK) and Brunauer-Emmett-Teller nitrogen adsorption analyzer (BET, TriStar Plus, Micromeritics, Norcross, GA, USA). Detailed information [41,42,43] about these methods is presented in the Supporting Information (Section SA).

### 2.3. Chemical Activation of Chitin-Based Carbons with NaOH

The activation of the chitin-based carbons by NaOH (>98% anhydrous, Sigma-Aldrich, St. Louis, MO, USA) was performed based on the protocol presented by Cazetta et al. [44] with a ratio of NaOH:raw material equal to 3:1. The NaOH:raw material was placed in the horizontal oven at a rate of 5 °C min^−1^ from room temperature to 1000 °C and kept for 1 h. In this process, the carbonization of the chitin materials was performed at the same time as the NaOH activation. 

### 2.4. Electrochemical Studies

The electrochemical studies were assessed through the method previously presented by Brandão et al. [36]. Detailed information on the preparation of the DES electrolyte, as well as the preparation/coating of the glassy carbon electrode with the chitin-derived biocarbon, are presented in Supplementary Information (Section SA) followed by the electrochemical characterization of the half-cell setup, in which the parameters considered to perform cyclic voltammetry, galvanostatic charge/discharge curves, and electrochemical impedance spectroscopy (EIS, Methrom, Herisau, Switzerland) are detailed.

The interfacial properties are highly dependent on temperature, as highlighted in a previous work performed by Brandão et al. [38]. The authors demonstrated that there is an increase in the specific capacitance with increasing temperature; however, a less favorable effect may occur related to a reported decrease in the capacitance retention probably due to the decrease in electrolyte viscosity. Further, ethaline crystallizes at T~20 °C, and therefore setting the electrochemical cell at 30 °C is a compromise between the need to protect the electrolyte structural integrity vs. lowering the system capacitance retention, which degrades the capacitor performance.

## 3. Results

Chitin-derived carbons from squids and prawns were thoroughly investigated by assessing the morphological analysis and electrochemical performance. Preliminary studies were performed taking into consideration different time intervals and temperatures of carbonization protocols, as presented in Table S1. The enhanced specific surface area and capacitance were obtained for the carbonization process performed at 1000 °C for 1 h. The mentioned parameters were selected for the extensive morphological characterization, cell design, and electrochemical testing presented and discussed in this chapter.

### 3.1. Structural Characteristics

**Figure 1:**
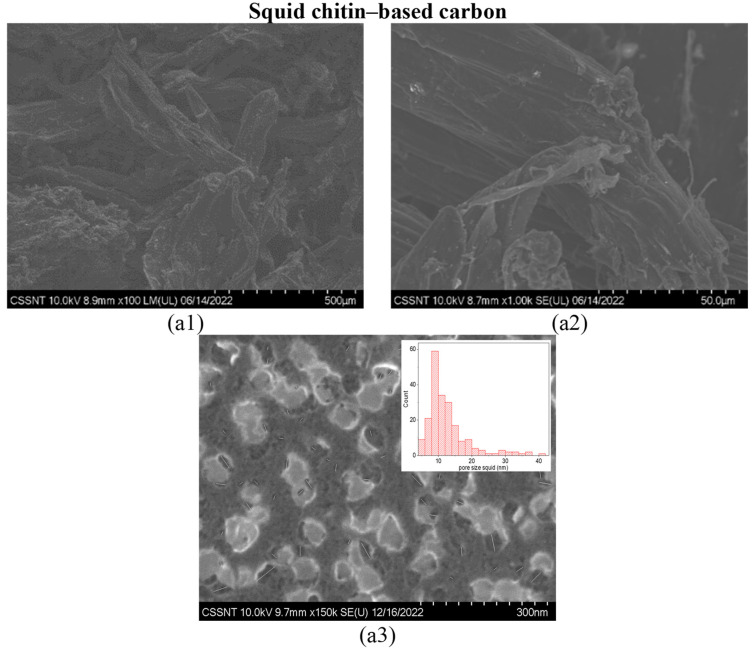
SEM images of squid chitin-based carbon (carbonized at 1000 °C for 1 h) at different magnifications: ×100 (**a1**), ×1 k (**a2**), and ×150 k (**a3**) with the pore size distribution in the inset of (**a3**).

**Figure 2:**
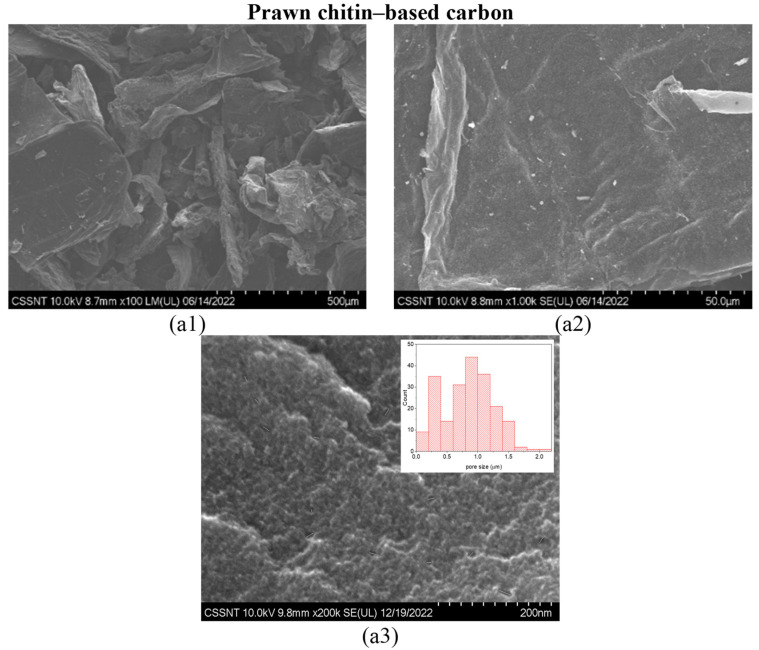
SEM images of prawn chitin-based carbons (carbonized at 1000 °C for 1 h) at different magnifications: ×100 (**a1**), ×1 k (**a2**), and ×200 k (**a3**) with the pore size distribution in the inset of (**a3**).

The SEM morphology of the squid and prawn chitin-derived carbon materials are shown in Figure 1 and Figure 2 at different magnifications of ×100, ×1 k, and ×150 k.

After the carbonization step for 1 h at 1000 °C, both chitin-derived biocarbons presented harsh morphology with well-defined graphene-like flakes. The histogram of the pore size distribution (~200 pores determined) was performed using the SEM image with ×150 k magnification and is represented in the inset of Figure 1(a3) and Figure 2(a3) for squid and prawn chitin-derived biocarbons, respectively. However, a more accurate study will be provided related to the determination of pore volume and surface area measured from the N_2_ adsorption/desorption isotherms (BET analysis).

The EDX analysis (Supporting Information, Figure S1a,b) shows, for both chitin-derived carbons, a high percentage of carbon (>90%) followed by Ca, O, Na, and K due to the organic nature of the raw chitin biopolymers.

### 3.2. XPS (X-ray Photoelectron Spectroscopy)

**Figure 3:**
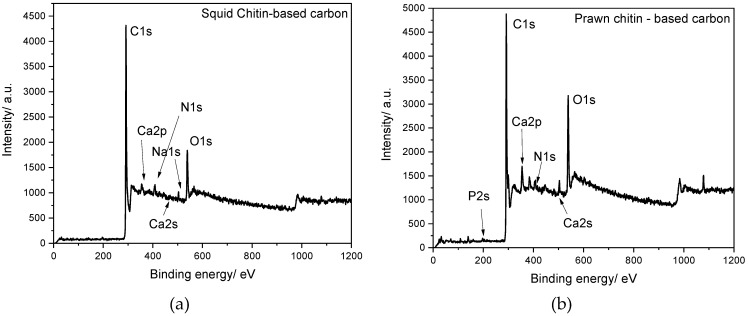
Overall XPS survey spectra for squid (**a**) and prawn (**b**) chitin-based carbon carbonized for 1 h at 1000 °C.

XPS was performed for a deeper understanding of the elemental analysis obtained from the EDX analysis. The XPS spectra of the chitin-derived carbons (carbonization protocol for 1 h at 1000 °C) are presented in Figure 3 in which the associated deconvoluted peaks for carbon (C1s), oxygen (O1s), and nitrogen (N1s) are fully presented in the Supporting Information, specifically in Figures S2 and S3 for squid and prawn chitin-derived carbons, respectively.

The results of the elemental analysis of the squid and prawn chitins-derived carbons are summarized in Table 1. Both carbon materials have high carbon content: 87.1% and 78.4% for squid and prawn chitins-based carbons, respectively.

The three predominant peaks at 284.9 eV, 529.9 eV, and 397.7 eV binding energies are related to C1s, O1s, and N1s, respectively [45]. These results showed that both samples hold carbon-, oxygen-, and nitrogen-containing functional groups in the structure of the carbonaceous material. Other components were also studied, such as Na1s, P2p, S2p, K2p, and Ca2p, with the number of N1s being almost identic in both samples. The remaining elements are also expected to be present in the assessed samples due to the organic sources of both raw chitin-based materials.

### 3.3. TEM (Transmission Electron Microscopy)

**Figure 4:**
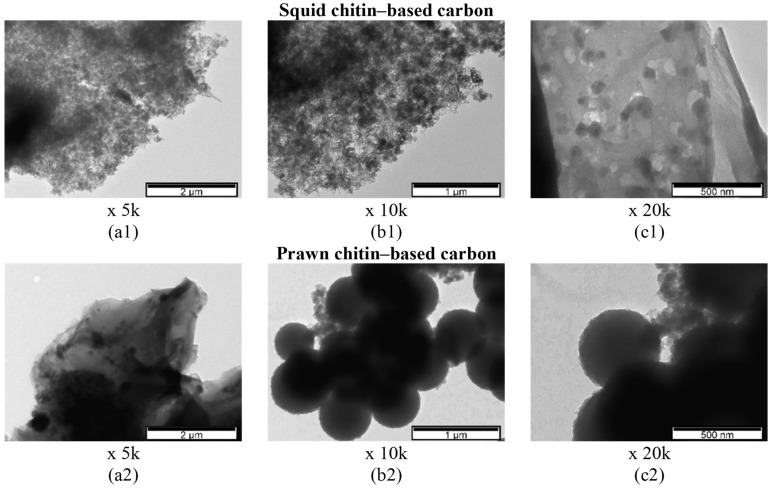
TEM images of squid and prawn chitin-based carbons (carbonized at 1000 °C for 1 h) at different magnifications: ×5 k (**a1**,**a2**), ×10 k (**b1**,**b2**), and ×20 k (**c1**,**c2**).

The detailed microstructure and morphology of both chitin–prawn and chitin–squid biocarbons were studied by TEM. The TEM images represented in Figure 4(a1,b1,c1), obtained at several magnifications for squid chitin-based carbon, show a 3D porous network, while for the prawn chitin-based carbon (Figure 4(a2,b2,c2)), the morphology seems to be distinct, revealing darker, well-defined circles, which may be associated with calcium, as demonstrated in the EDX analysis (Supporting Information Figure S1a). The squid chitin-based carbon presents nanosheets that present a transparent, silk-like, folded morphology, which may indicate an ultrathin nature.

### 3.4. ATR-FTIR and Raman Spectra Analysis

**Figure 5:**
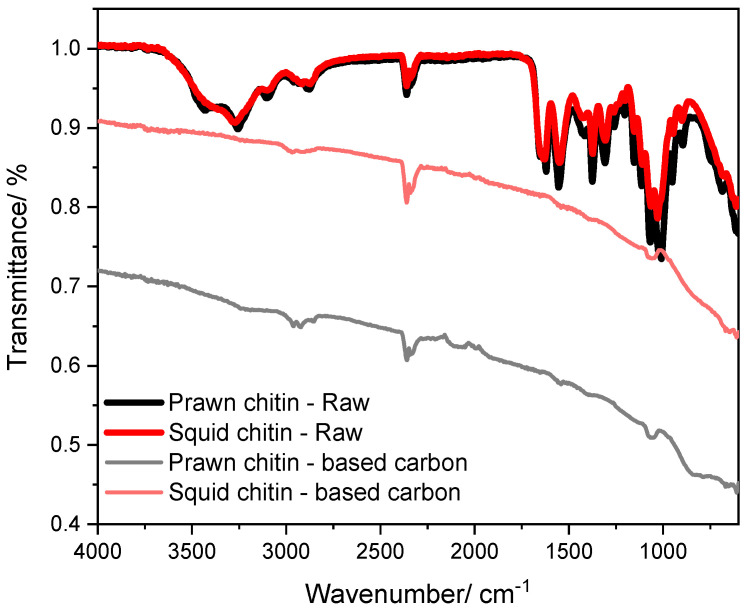
ATR-FTIR spectra of raw prawn and squid chitin carbon precursors, as well as the chitin-based carbons (1000 °C, 1 h).

The ATR-FTIR spectra of prawn and squid chitins-based carbons obtained in the range of 4000–750 cm^−1^ are presented in Figure 5. A comparison is made between the raw squid and prawn chitins (before the carbonization process) and the chitin biocarbon-based materials. The raw prawn and squid chitins spectra show a distinctive and large band between 3500 and 3250 cm^−1^ assigned to the stretching vibrations of the hydroxyl (-OH) groups, which are no longer present after the carbonization process. Around 1000 cm^−1^, a high-intensity band is also present, which represents the stretching vibration of the C-O group. The functional groups containing oxygen promote the hydrophilic properties of the surface. In addition, the double band reported between 1089 and 1043 cm^−1^ in the spectrum of the raw prawn and squid chitins belongs to the =C-O-C band vibration in the aromatic ether [46]. The same band is noticeable in both carbonized samples, however, with a lower intensity. In the chitin-based biocarbon samples, the band observed between 2900 and 2800 cm^−1^ can be assigned to the C-H bands of aliphatic hydrocarbons, which present low intensity [47,48]. The peak around 2350 cm^−1^ results from the C=C stretching vibrations, which can be associated with CO_2_ [49]. 

**Figure 6:**
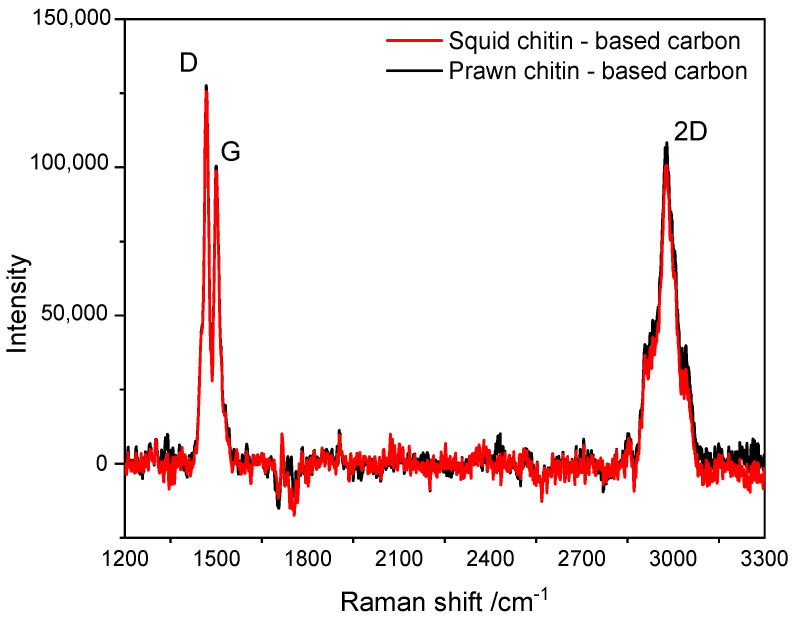
Raman spectra of the chitin-based carbons.

The Raman spectra measured for the raw prawn and squid chitins-based carbons are presented in Figure 6. The analysis of the first and second Raman regions are fully presented in Supporting Information Figure S4a,b. The first Raman region has been deconvoluted using a Gaussian fit. The D band (D_1_ is presented in Figure S4a) occurred around 1470 cm^−1^, and it may be associated with structural defects combined with a disordered carbon structure. The G band at 1510 cm^−1^ is attributed to the structural graphitic order of the material [50,51].

The ratio between the intensity of these two characteristic bands (ratio: I_D_/I_G_) is presented in Table 2. The results show that the prawn chitin-based carbon presents a slightly higher ratio I_D_/I_G_ (1.68) compared to the squid chitin-based carbon (1.59) with a crystallite size of 12 and 11 nm, respectively. A decrease in I_D_/I_G_ may be associated with an increase in the graphitization degree [52]. However, the similarity found in these values may indicate that both samples present insignificant changes regarding their carbon bulk structure.

To the best of the authors’ knowledge, no other works reported in the literature correlated the Raman characterization of chitin-based biocarbon materials with the material parameters obtained by BET analysis (surface area, pore size, etc.). The change in crystallinity of the two studied samples may lead to a change in the physical properties of the biocarbon, while the increase in crystallinity may be correlated to the boost reported in the capacitance value [53]. Li et al. [54] prepared a carbon aerogel from crab shell-derived chitin nanofibers in which the material carbonized at 900 °C presented an I_D_/I_G_ value of 0.987, much lower if compared to the values presented in this work. Nevertheless, it is crucial to consider the fact that the biocarbon source is distinct in both works, which may lead to different carbon structure materials.

### 3.5. XRD Analysis 

**Figure 7:**
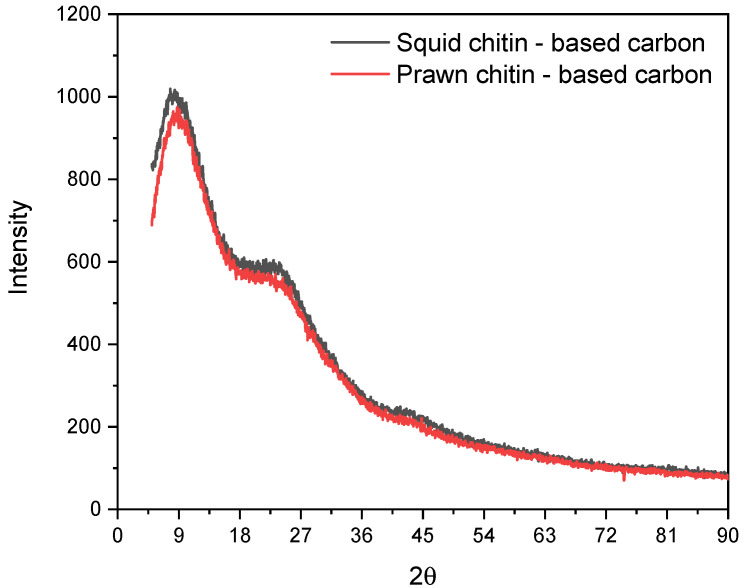
X-ray diffraction (XRD) patterns for squid (black) and prawn (red) chitins-based carbons.

Figure 7 shows the XRD patterns of the squid and prawn chitins-based carbons. The squid and prawn chitins-based biocarbon samples exhibit similar X-ray diffraction patterns.

Both samples are amorphous and characterized by broad reflection peaks at around 8, 24, and 45°. The peaks associated with 2θ = 24 and 44/45° correspond to the (002) and (100) planes of graphitic carbon, respectively [55,56]. The wide (002) diffraction pattern portrays the existence of parallel-stacked graphene sheets in the solid solution of carbon materials [21]. Likewise, the presence of the (100) plane reveals the existence of sp^2^ hybridized carbon with a honeycomb structure, which could improve the conductivity of the material [57,58], thus is expected to affect the electrochemical properties and anatomy of the biocarbon composite electrodes/electrolyte interface.

Table 3 summarizes the peaks extracted from the diffraction pattern obtained for the squid and prawn chitin-based carbons (carbonization protocol for 1 h at 1000 °C). For the squid chitin-based carbon, the (100) plane presents a much higher intensity compared to the prawn chitin-based carbon, meaning that the squid involves a higher density of pores inside the solid-state graphitic carbon [59,60]. The interlayer spacing calculated from the (002) plane is 3.7 Å for squid and 3.6 Å for prawn chitin-based carbon, which are higher than the 3.35 Å presented for pure graphite [61]. The chitin-based carbon materials seem to present good quality and a high potential for their application as electrodes in supercapacitors devices.

### 3.6. BET Analysis 

**Figure 8:**
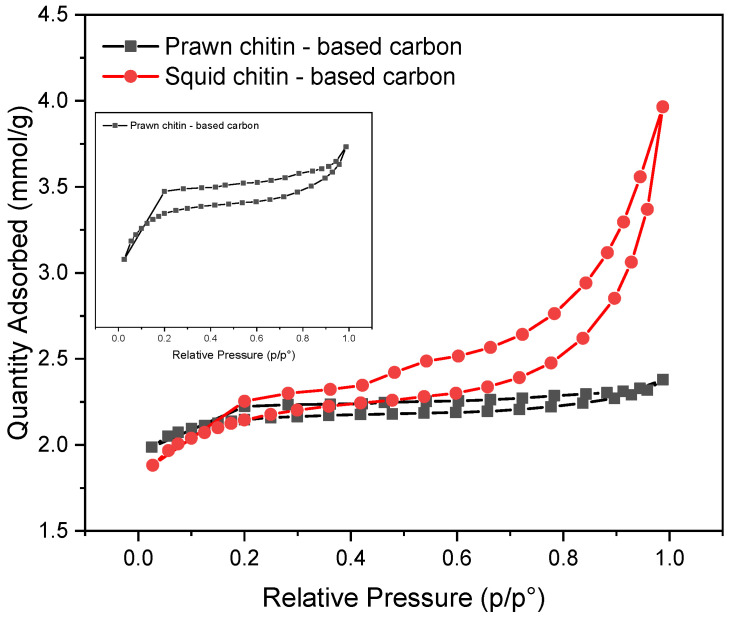
N_2_ adsorption–desorption isotherms for prawn and squid chitin-based carbons carbonized for 1 h at 1000 °C.

Figure 8 shows the N_2_ adsorption/desorption isotherms for prawn and squid chitin-based carbons. Both samples present isotherms classified as the Type IV class according to the Brunauer, Deming, and Teller (BDT) classification [62]. 

The main features of the isotherms displayed in Figure 8 are its hysteresis loop, which is related to the capillary condensation taking place in the mesopores, and the limiting uptake over a range of high P/P_0_. Table 4 summarizes the BET surface area, microporous and mesoporous volume, as well as total pore volume, plus the mean pore diameter of the porous carbon samples. The BET analysis shows a surface area of 85.0 m^2^ g^−1^ and 149.3 m^2^ g^−1^, a total pore volume of 0.038 cm^3^ g^−1^ and 0.112 cm^3^ g^−1^, and a mean pore diameter of 8.47 nm and 7.12 Å for prawn chitin and squid chitin-based carbons, respectively, being both classified as micropores. Using the squid based-chitin as a carbon precursor leads to a higher surface area with an increase of almost ×2 compared to the prawn chitin precursor. 

### 3.7. Electrochemical Characterization

#### 3.7.1. Cyclic Voltammetry and Galvanostatic Charge–Discharge (GCD) Analysis 

**Figure 9:**
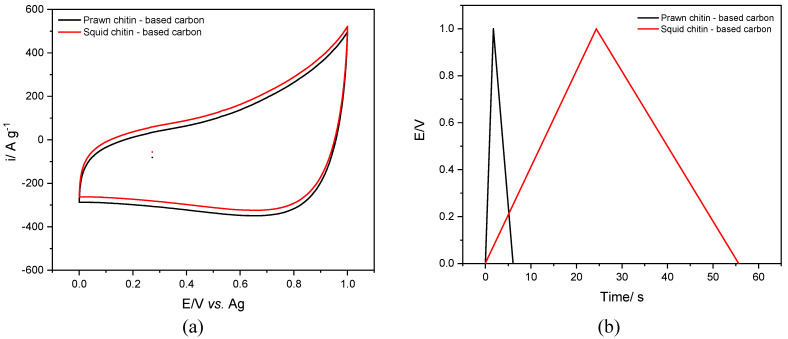
Electrochemical performance of the squid and prawn chitins-based biocarbon composite electrodes/DES (ethaline) interface in a three-electrode set-up, (**a**) cyclic voltammetry at 50 mV s^−1^, and (**b**) galvanostatic charge-discharge curves (GCD) recorded with current density 1 A g^−1^, at 30 °C.

The charge storage mechanism of electrified interfaces comprising high surface area carbon composite electrodes operated in a supercapacitor configuration is based on the electrochemical double layer formation at the interface between the electrode and the electrolyte, thus providing a large surface area for the adsorption and desorption of ions from the electrolyte. Cyclic voltammetry analysis was performed aiming to study the electrochemical performance of the chitin-derived biocarbon composite electrodes at the non-aqueous electrolyte ethaline interface, e.g., assess the electrochemical stability potential window (vs. a silver wire as a reference electrode). The voltammetric behavior of the squid and prawn chitins-based biocarbon is presented in Figure 9a, at a scan rate of 50 mV s^−1^. 

Both biocarbon materials composite electrodes displayed quasi-rectangular voltammetric profiles, indicating a characteristic electric double-layer capacitive behavior [63]. There is a slight displacement in the current response in the squid chitin-based carbon, compared to the prawn chitin-based carbon, possibly due to differences in the chemical composition, surface structure, or morphology of the biocarbon materials (degree of graphitization or porosity).

Overall, the cyclic voltammogram shape obtained by the two carbon materials provides insight into their unique electrochemical properties, which can be useful for designing and optimizing materials for various applications, such as energy storage devices.

Charge-discharge curves obtained from high-specific area materials in a supercapacitor operation provide important information about the electrochemical performance of the electrode material and cell design, thus allowing to establish a relationship between the electrode potential and the amount of charge stored or released during the charging and discharging processes. 

The charge-discharge curves obtained from high-specific area materials such as biocarbon derived from marine waste in a supercapacitor operation may provide important information about the electrochemical properties and performance of the electrode material, which is crucial for designing and optimizing supercapacitor systems for various applications. By analyzing Figure 9b, the measurements were taken under the same conditions, such as temperature, voltage range, current density, and electrolyte composition in which only the material nature of the composite electrode was varied. The GCD curves for the squid and prawn chitins-based biocarbon composite electrodes present a symmetric triangle shape in both materials with distinct charge and discharge time scales, although evidencing the electric double-layer capacitor behavior and the high reversibility of the carbon electrodes, their performance is distinct [64]. The prawn chitin-derived biocarbon presented faster charge-discharge times compared to the larger surface area squid chitin biocarbon (~9× slower). High-specific area materials have a larger surface area per unit mass, which means that they can store more charge and release it more efficient compared to low-specific area materials.

Table 5 shows the specific capacitance and the capacitance retention calculated from the GCD curves of squid and prawn chitins-derived biocarbon composite/ethaline interface after 1000 and 5000 cycles. The higher specific capacitance was obtained for the larger surface area sample squid chitin-based biocarbon, at a current density of 1 A g^−1^. The capacitance retention presented values of 95.7% and 92.1% for squid and prawn chitin-based carbons, respectively, after 1000 cycles, with a further decrease to 93.3% and 84.1%, respectively, after 5000 cycles. The higher specific capacitance (20 F g^−1^) value can be associated with the higher specific surface area of 149.3 m^2^ g^−1^ and larger micropore volume of 0.112 cm^3^ g^−1^ estimated for the squid chitin-based carbon. The larger surface of a high specific area biocarbon material can accommodate more electrolyte ions, which results in a greater amount of stored charge. Furthermore, the squid chitin-based carbon high specific area is accompanied by a highly porous structure with a large volume of pores, which can also contribute to the higher specific capacitance. These pores can provide additional surface area for charge storage and facilitate the diffusion of ions into and out of the electrode. The ability of electrolyte ions to penetrate the pores of biocarbon-based materials is a key factor that affects their performance in storage devices.

The relationship between the increase of the specific capacitance with the increase of the specific surface area of the material was already reported by Brandão et al. [36]. Other authors also established the correlation between the increase in surface area and pore volume which leads to more active surface sites that are available for charge accumulation [65,66,67].

#### 3.7.2. EIS Analysis

**Figure 10:**
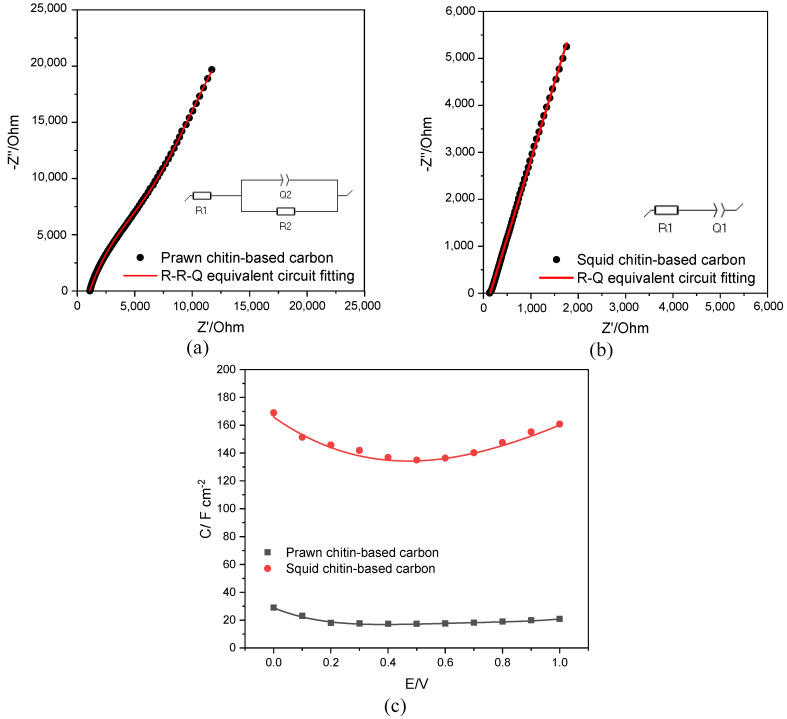
Nyquist plots for prawn (**a**) and squid (**b**) chitin-based carbons (+0.5 V vs. Ag). R(RQ) and RQ fitted to experimental data. (**c**) differential capacitance (F cm^−2^) curves measured at chitin-prawn and chitin-squid/DES interface in the accessible potential window (0–1 V).

Figure 10a,b show the Nyquist (Z″ vs. Z′) plots measured at the fixed potential at 0.5 V (vs. Ag) for GC coated with prawn and squid chitins-based biocarbon composite electrode/DES electrolyte interface. To simplify, and to demonstrate the quality of the fitting it is presented the Nyquist plots measured at +0.5 V (as an example). For both chitin-prawn and chitin-squid composite electrodes, the equivalent circuit considered for the fitting was different (see inset Figure 10a,b). For the prawn chitin-based biocarbon, an R(RQ) equivalent circuit was fitted the experimental results in the whole accessible electrochemical window, while for the squid chitin-based biocarbon, an RQ equivalent circuit allowed obtaining an acceptable χ^2^ (the quality of the fitting was judged by the value of χ^2^ below 10^−3^). The parameters of the circuit elements are summarized in Tables S2 and S3, in the Supporting Information, Section SD. The equivalent circuit fitting to Nyquist diagrams allowed the estimation of the differential capacitance (F cm^−2^) from 0 to 1 V accessible window, and it is represented in Figure 10c. The capacitance-potential dependence curves present a U-shape for both electrified interfaces, as described in the literature [36,68,69]. Comparing the marine waste source of the biocarbon, a higher capacitance was obtained for squid chitin-based carbon, corroborated by the galvanostatic charge-discharge curves profile. However, an increase of up to ×80, between the two carbon materials, is reported if considered the EIS analysis, much higher compared with the difference found from capacitance estimated considering the galvanostatic charge-discharge curves method.

### 3.8. Preliminary Studies: Carbon Activation and Aqueous Electrolytes Effect on the Capacitance

#### 3.8.1. Chitin-Based Carbon Activation with NaOH

For a better understanding of how the characteristics of these materials can be enhanced through an activation process, preliminary electrochemical studies using NaOH as an activating agent (chitin-based carbon-NaOH/DES electrolyte) were assessed.

The activation process was performed in the oven at 1000 °C for 1 h, the same procedure used for the carbonization of the chitin raw materials. According to Lillo-Ródenas et al. [70], the reaction between NaOH and carbon begins at around 570 °C. The surface area was again investigated by BET analysis, showing a slight increase of both surface area and pore volume in the two carbons after activation with NaOH, as seen in Table 6. 

**Figure 11:**
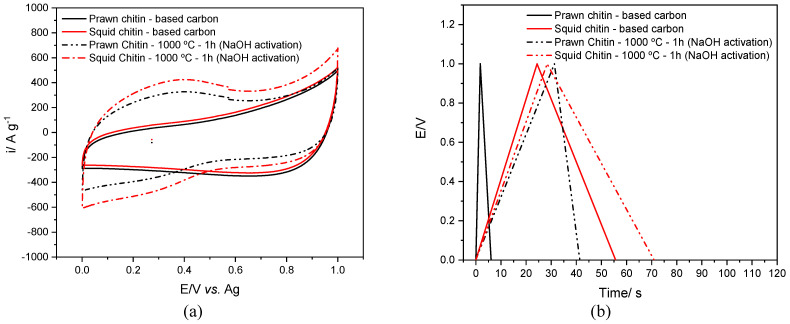
Electrochemical study of the squid and prawn chitins-based biocarbon (with and without NaOH activation) in DES electrolyte (ethaline), (**a**) cyclic voltammetry at 50 mV s^−1^, and (**b**) galvanostatic charge–discharge curves recorded with current density 1 A g^−1^ at 30 °C.

Figure 11a presents the cyclic voltammetry, and Figure 11b presents the charge–discharge curves of the activated carbons compared with the raw chitin-based carbon materials. 

The results point to a change in the structure of the biocarbon probably due to the addition of -OH functional groups, which may help to enhance its characteristics, particularly their specific capacitance. In the present study, it increased from 20 to 32 F g^−1^ for the squid chitin- and from 15 to 29 F g^−1^ for the prawn chitin-based biocarbon before and after the activation step, as highlighted by the values summarized in Table 7. 

The capacitance retention of the activated chitin-based carbons presents lower values compared to the nonactivated carbons for 1000 and 5000 cycles. Even though there is an increase in capacitance with the activation process, that is not observed with the capacitance retention over cycles.

Table 8 summarizes the best results presented in the literature compared with the results obtained in this work. It is difficult to make a straightforward comparison of the available data since the surface area of the carbon materials strongly depends on the origin of the raw material and the extraction treatment. Nevertheless, the capacitance data depends on the experimental setup used and on the active mass of the biocarbon material considered to construct the composite electrode, which will certainly influence the electrode area; however, for most of the work found in the literature, it is a complex task to obtain this data in its full extent to perform a proper comparison. Gao et al. [22] fabricated N-doped activated carbons considering a demineralization–deproteination–deacetylation–activation process of the prawn shell of “Bohai prawn”. Kasprzak et al. [71] obtained chitin from shrimp shells, and Justin Raj et al. [19] obtained chitin from the squid gladius separated from squid fish. Even though the results presented in this work are significantly lower than those obtained in the literature (lower specific surface area and consequent capacitance), it must be considered that the carbon source differs (i.e., chitin precursors), and it is extremely difficult to make comparisons when natural products with large variability are involved. Furthermore, the purification method, as well as the preparation/carbonization method, cannot be fully compared. The preparation method used in this work is unique since the deproteinization method is based on enzymatic hydrolysis instead of chemical hydrolysis as commonly presented in the literature. Additionally, different surface areas can lead to varying physical and chemical properties, such as porosity, electrical conductivity, and chemical reactivity. This is strictly related to the specific capacitance obtained for each carbon in contact with the electrolyte.

#### 3.8.2. Aqueous Electrolytes’ Effect on the Capacitance

Since the squid chitin-based carbon presents the best performance, this material was the one selected to pursue the study of the two aqueous electrolytes without any previous activation. The electrochemical analysis of the squid chitin-based carbon with aqueous electrolytes was performed, and the results are summarized in Table S4, Figure S5 (presented in Supporting Information, Section SE), and also in Table 8. Compared to the use of ethaline, the use of 1 mol L^−1^ H_2_SO_4_ electrolyte presents a slight increase in specific capacitance, and a decrease is reported when using 1 mol L^−1^ KOH electrolyte, as described in Table 8. The major difference, however, is observed in capacitance retention. The eutectic electrolyte presents adequate capacitance retention for applications in supercapacitors (~96%) after 1000 cycles. Both aqueous electrolytes present lower capacitance retention rates after 1000 cycles (~86% for H_2_SO_4_ and ~80% for KOH) and after 5000 cycles (~45% for H_2_SO_4_ and ~33% for KOH). The lower retention rates estimated in aqueous electrolytes are strictly related to the viscosity of the electrolyte when a decrease in viscosity may lead to a decrease in capacitance [72]. Several studies [73,74,75] were performed for biomass-based carbons using both 1 mol L^−1^ H_2_SO_4_ and KOH electrolytes, presenting specific capacitances around 200 F g^−1^ and capacitance retentions around 95%. In all these studies, the electrode preparation was made through a polymer-based carbon slurry in a two-electrode setup with a higher mass of active material, leading to higher capacitance and retention when comparing aqueous electrolytes.

## 4. Conclusions

A simplistic and sustainable protocol strategy to prepare porous biocarbon materials from chitins (extracted from squid and prawn waste products) was proposed. In this work, porous carbon materials were obtained with a 1-step carbonization process for 1 h at 1000 °C without any further chemical activation treatment. The squid and prawn chitins-based biocarbon materials present naturally doped nitrogen atoms, delivering a specific capacitance of 20 and 15 F g^−1^ at 1 A g^−1^, respectively, along with capacitance retention up to ~96% after 1000 continuous charge–discharge cycles.

Preliminary results regarding the effectiveness of the biocarbon activation with NaOH and different aqueous electrolytes instead of eutectic mixtures were tested, which led to an increase in capacitance with the activation with NaOH using ethaline as the electrolyte compared to considering a 1 mol L^−1^ H_2_SO_4_ electrolyte. Further studies are required for a deeper understanding of the impact of material activation on the interfacial structure and performance. However, the decrease in the capacitance retention rate makes the nonactivated carbons/DES structures suitable candidates for practical electrochemical applications.

The chitin-based biocarbon prepared in this work was tested with and without any chemical activation and in contact with an eco-friendly choline chloride-derived electrolyte. Even though these results present a lower surface area and lower capacitance, compared to other published results, it is an important insight that chitin prawn/squid biocarbon is suitable to replace carbon electrode materials derived from fossil fuel sources in supercapacitors alongside an eco-friendly electrolyte to bring more effective and sustainable energy storage devices. The future of chitin-based biocarbon composite electrodes for supercapacitor applications appears to be promising; nevertheless, the advance is still reliant on important advances toward the improvement of their electrochemical performance and consequently the upscaling step. However, challenges still need to be addressed to develop protocols to produce competitive marine waste-derived biocarbon compared with conventional materials to produce electrodes. Additionally, further interdisciplinary research in materials science, chemistry, and energy storage is required to understand the full potential of chitin-based carbons and their impact on the energy storage industry.

## Figures and Tables

**Table 1:** At% composition of the elements presented in the squid and prawn chitins-based carbons.

	At%			
	Chitin-Based Carbons		Raw Material	
Element	Squid Chitin	Prawn Chitin	Squid Chitin	Prawn Chitin
C1s	87.1	78.4	59.8	51.4
N1s	2.2	2.1	0.91	1.1
O1s	9.0	14.3	37.5	43.4
Na1s	0.3	0.7	-	-
P2p	0.3	0.8	0.52	0.5
S2p	0.06	0.05	-	-
K2p	0.5	2.3	0.62	2.4
Ca2p	0.6	1.4	0.65	1.2

**Table 2:** I_D_/I_G_ and crystallite size values of the prawn and squid chitin-based carbons through the peak deconvolution of the Raman spectra in the first Raman region.

Carbon Source	R^2^	I_D_/I_G_	La/nm
Prawn chitin	0.99	1.68 ± 0.01	12
Squid chitin	0.98	1.59 ± 0.02	11

**Table 3:** Peaks are extracted from the diffraction pattern that is represented in Figure 7.

	No.	2θ (Deg)	d (Å)	FWHM (Deg)	Int. I (Counts Deg)
Squid chitin-based carbon	1	8.2	10.6	9.6	4579.1
	2	24.1	3.7	9.6	563.5
	3	44.7	2.0	9.6	462.3
Prawn chitin-based carbon	1	9.0	9.8	0.1	66.5
	2	24.4	3.6	0.1	43.1
	3	43.7	2.1	0.1	7.1

**Table 4:** BET surface area parameters of the chitin-based carbon materials.

BET Isotherms Analysis					
Carbon Source	S_BET_ (m^2^ g^−1^)	V_micro_ (cm^3^ g^−1^)	V_meso_ (cm^3^ g^−1^)	V_total_ (cm^3^ g^−1^)	D_p_ (Å)
Prawn chitin	85.0	0.029	0.009	0.038	8.47
Squid chitin	149.3	0.053	0.059	0.112	7.12

**Table 5:** Specific capacitance and % retention after 1000 and 5000 cycles of squid and prawn chitins-based carbons samples (1 A g^−1^).

	Carbonization		Electrochemistry 30 °C Ethaline		
Carbon Precursor	Temperature/°C	Time/h	C/F g^−1^ 1st Cycle	% Retention after 1000th Cycle	% Retention after 5000th Cycle
Squid Chitin	1000	1	20 ± 1	95.7	93.3
Prawn Chitin			15 ± 2	92.1	84.1

**Table 6:** BET surface area parameters of the chitin-based carbon materials with and without activation procedure.

BET Isotherms Analysis					
Carbon Source	S_BET_ (m^2^ g^−1^)	V_micro_ (cm^3^ g^−1^)	V_meso_ (cm^3^ g^−1^)	V_total_ (cm^3^ g^−1^)	D_p_ (Å)
Prawn Chitin_NaOH activation	86.2	0.021	0.011	0.032	1.110
Squid Chitin_NaOH activation	154.2	0.066	0.071	0.137	0.981

**Table 7:** Specific capacitance and % retention after 1000 and 5000 cycles of squid and prawn chitins-based biocarbon composite electrodes (with and without NaOH activation) (1 A g^−1^) in ethaline.

	Carbonization		Electrochemistry 30 °C Ethaline		
Carbon Precursor	Temperature/°C	Time/h	C (F g^−1^) 1st cycle	% Retention after 1000th Cycle	% Retention after 5000th Cycle
Squid Chitin_NaOH activation	1000	1	32 ± 4	93.9	88.1
Prawn Chitin_NaOH activation			29 ± 3	90.2	79.4

**Table 8:** Literature review of chitin-based porous carbon electrodes from fish/marine waste for supercapacitors.

Marine Waste Source	Material	Configuration	Surface Area m^2^ g^−1^	Electrolyte	Current Density A g^−1^	Capacitance F g^−1^	Capacitance Retention %	Reference
Squid chitin (*Illex* *argentinus*) byproducts from the industrial processing	Porous carbon	3 electrode- cell (Active mass: 4 mg) (electrode diameter: 4 mm)	149	Liquid DES	1	20 ± 1	96 @ 1000 cycles/93.3 @ 5000 cycles	This work
	Porous carbon NaOH activation		154			32 ± 4	93 @ 1000 cycles/88.1 @ 5000 cycles	
	Porous carbon		149	1 mol L^−1^ H_2_SO_4_		23 ± 2	86 @ 1000 cycles/45 @ 5000 cycles	
			149	1 mol L^−1^ KOH		12 ± 3	80 @ 1000 cycles/33 @ 5000 cycles	
Prawn chitin (*Penaeus* *vannamei*) byproducts from the industrial processing	Porous carbon		85	Liquid DES		15 ± 2	92 @ 1000 cycles/84.1@ 5000 cycles	
	Porous carbon NaOH activation		86			29 ± 3	94 @ 1000 cycles/79.4 @ 5000 cycles	
Prawn shells “Bohai prawn”	N- activated carbon	2 electrode “sandwich” cell (Active mass: 3–4 mg)	1918	1 mol L^−1^ H_2_SO_4_ 6 mol L^−1^ KOH	0.05	695 357	95 @ 5000 cycles	[22]
Shrimp shells chitin (α-Chitin from BioLog)	Porous carbon	Swagelok^®^-type cell (3-electrode–cell) (electrode diameter: 11.8 mm)	1298	1 mol L^−1^ Li_2_SO_4_	15	142	92	[71]
Gladius of Squid fish (*Todarodes pacificus*)	N- and O- activated carbon	Stainless-steel split test cell (EQ-STC) (2 electrode–cell) (Active mass: 2.5 mg)	1129	1 mol L^−1^ H_2_SO_4_	1	204	100 @ 20,000 cycles	[19]

## Data Availability

The data obtained is presented in Supporting Information in the Origin files, where the results are available.

## Supplementary Materials

The following supporting information can be downloaded at: https://www.mdpi.com/article/10.3390/ma16062332/s1, SA. Materials and Methods; SB. Carbonization parameters; SC. Figures; SD. Elements of the equivalent circuits for the EIS analysis; SE. Preliminary results–electrochemical studies; SF. References. Figure S1. EDX analysis of squid (a) and prawn (b) chitin-based carbons, Figure S2. Deconvolution of the peaks of the XPS survey spectra for squid chitin-based carbon carbonized for 1 h at 1000 °C: C1s (a), O1s (b), and N1s (c). Figure S3. Deconvolution of the peaks of the XPS survey spectra for prawn chitin-based carbon carbonized for 1 h at 1000 °C: C1s (a), O1s (b), and N1s (c). Figure S4. First Raman region (a) and second Raman region (b) of the Raman spectra of the chitin-based carbons. Figure S5. Electrochemical study of the squid chitin-based carbon in DES, 1 mol L^−1^ H_2_SO_4_, and 1 mol L^−1^ KOH (a) cyclic voltammetry at 50 mV s^−1^ and (b) galvanostatic charge–discharge curves recorded with current density 1 A g^−1^ at 30 °C. Table S1. Carbonization time and temperature for prawn and squid chitin with associated SBET, capacitance, and % retention. Table S2. Parameters of all elements of the equivalent circuit (RRQ) for prawn chitin-based carbon, Table S3. Parameters of all elements of the equivalent circuit (RQ) for squid chitin-based carbon, Table S4. Specific capacitance and % retention after 1000 and 5000 cycles of squid chitin-based carbons sample (1 A g^−1^) in DES, 1 mol L^−1^ H_2_SO_4,_ and 1 mol L^−1^ KOH.
