# Supplementary material for: Chitins from Seafood Waste as Sustainable Porous Carbon Precursors for the Development of Eco-Friendly Supercapacitors
Source: Materials (Basel). 2023 Mar 14;16(6):2332. doi: 10.3390/ma16062332 (PMC10057302; doi:10.3390/ma16062332)
Supplement: Supplementary file 1 [file materials-16-02332-s001.zip › materials-2239609-supplementary.pdf]

Supporting Information

Chitins from Seafood Waste as Sustainable Porous Carbon Precursors for the Development of Eco-Friendly Supercapacitors

Ana T. S. C. Brandão [1], Renata Costa [1], Sabrina State [2], Pavel Potorac [2], Catarina Dias [1], José A. Vázquez [3], Jesus Valcarcel [3], A. Fernando Silva [1], Marius Enachescu [2,4] and Carlos M. Pereira [1,*]

1 Instituto de Ciências Moleculares IMS-CIQUP, Departamento de Química e Bioquímica, Faculdade de Ciências da Universidade do Porto, Rua do Campo Alegre, 687, 4169–007 Porto, Portugal
2 Center for Surface Science and Nanotechnology, University Polytechnica of Bucharest, Splaiul Independentei, 313, 060042 Bucharest, Romania
3 Grupo de Reciclado y Valorización de Residuos (REVAL), Instituto de Investigaciones Marinas (IIM-CSIC), 36208 Vigo, Spain
4 Academy of Romanian Scientists, Splaiul Independentei 54, 050094 Bucharest, Romania
* Correspondence: cmpereir@fc.up.pt

This supplementary file includes the following:

- Morphological characterization of the chitin-based carbon materials and electrochemical studies related to the Materials and Methods section of the main text.
- Preliminary results associated with the activation of the chitin-based carbons and the electrochemical studies using aqueous electrolytes.

SA. Materials and Methods

- **Morphological characterization**

The morphology of the chitin-based carbon samples was assessed by scanning electron microscopy (SEM) associated with energy-dispersive X-ray (EDX, Oxford detector-analyzer) analysis (Hitachi SU 8230 equipment (Tokyo, Japan)).

Transmission electron microscopy (TEM) (Hitachi model H8100, with LaB6 filament and using an accelerating voltage of 200 kV) was also used.

Raman spectra was recorded with Raman spectrometer Ramos PA532 Ostec, using a 532 nm excitation wavelength. In addition, the crystallite size of the sp_2 lattice (La) was calculated, according to the equation presented by Pimenta et al. [41]:

$$L_a = 2.4 \times 10^{-10} \lambda_L^4 \left(\frac{I_D}{I_G}\right)^{-1} \qquad \text{(S1)}$$

where λ_L is the excitation wavelength, I_D/I_G is the ratio between the intensity of the D and G bands.

X-ray diffraction measurements of all samples were carried out in the 5 – 90° range on a Rigaku Smart Lab X-Ray Diffractometer (Rigaku Corporation, Tokyo, Japan) using Cu Kα radiation (λ=0.154060 nm) operating at room temperature. The identification of the phase was made by referring to the International Center for Diffraction Data – ICDD (PDF-2) database.

The chemical composition of the obtained materials was tested by Kratos Axis Ultra HSA X-ray Photoelectron Spectroscopy (XPS) system (Kratos Analytical Ltd, Manchester, UK), with a 15 kV X-ray source. Attenuated total reflectance infrared (ATR-FTIR, Bruker FT-IR System Tensor 27 spectrophotometer (Massachusetts, USA)) measurements were performed using a Bruker FT−IR System Tensor 27 spectrophotometer (Massachusetts, USA) in the range of 4000 to 600 cm^{-1}.

- **Electrochemical studies**
  - Preparation of DES electrolyte

Choline chloride (ChCl, Sigma-Aldrich, 99%) was dried at 60ºC in the oven overnight before use, and ethylene glycol (Sigma-Aldrich, 99%) was used as received. The eutectic mixture was prepared by stirring and heating at 60 ºC the ChCl with ethylene glycol, as HBD, in the molar ratio of 1:2, until a homogeneous and clear liquid was formed. This liquid will be referred from now on as ethaline.

  - Aqueous electrolytes

Alongside the DES preparation, two aqueous electrolytes (1 mol L^{-1}) were also prepared, H_2SO_4 (95%, Sigma-Aldrich) and KOH (pellets, 98%, Sigma-Aldrich).

  - Glassy Carbon (GC) electrode preparation

The immobilization of the carbon material (squid and prawn chitin-based carbons) on the electrode was successfully achieved, using the method previously described by Brandão *et al.* [36]. Briefly, a dispersion of 5 mg of carbon in 950 μL N, N-Dimethylformamide (DMF, 99.8%, Sigma Aldrich) and 10 μL Nafion® 117 (~5 %, Sigma Aldrich) was prepared. Ultrasonication was used for a period of 2 h, to obtain a homogeneous dispersion of the material. The weight of the carbon-coated on the GC electrode was obtained as an average of several measurements, taking into consideration that the material and the Nafion dispersion in DMF is homogeneous. The suspension was drop cast on the GC electrode surface using a micropipette and was dried at room temperature before combining it with the electrochemical cell and immersed in the eutectic mixture.

- Electrochemical characterization of half-cell setup

The electrochemical measurements were performed in a three-electrode cell using a computer-controlled VSP-300 multichannel potentiostat from BioLogic, controlled with EC-Lab V11.26 software.

The three-electrode configuration consisted of a GC electrode as a working electrode, a graphite counter electrode, and a silver wire pseudo-reference electrode. The working electrode was polished following the method previously presented by several authors [36,38,42].

The experiments were performed at 30 °C (using a water jacket three-electrode electrochemical cell and the LTD-6 Grant thermostatic water bath (Grant Instruments, UK) to control the electrolyte temperature) for both eutectic and aqueous electrolytes. Voltammetric investigations were carried out at 50 mV s^{-1}, starting at 0 V towards the positive side up to 1 V.

Galvanostatic charge/discharge curves were obtained at a current density of 1 A g^{-1}. The specific capacitance in a three-electrode configuration was calculated from the galvanostatic discharge curves using **Equation S2**, according to Stoller *et al.* [43]:

$$C = \frac{I\,\Delta t}{m\,\Delta V} \qquad \text{(S2)}$$

where I is the discharge current (A), Δt is the discharge time (s), ΔV is the potential window (V), and m is the weight of the carbon material in the electrode (g).

The differential capacitance (F cm^{-2}) can be obtained from the electrochemical impedance spectroscopy (EIS) measurements at fixed potentials from 0 V to 1 V (0.1 V intervals).

Nyquist spectra were collected applying a sinusoidal signal of 10 mV (RMS) and with frequencies logarithmically distributed in the range of 20 kHz to 1 Hz.

The extraction of the capacitance can be obtained from the Z″ – Z′ plot, from the parameters of CPE (Y_0, n) obtained from the adjustment of the equivalent circuit R-Q, according to **Equation (S3)**, and R-(R-Q), according to **Equation (S4)**, in which the presence of a slight curvature on the impedance Nyquist plot [36]. The quality of the fitting was judged by the value of χ^2 ($<10^{-3}$).

$$C = Y_0^{1/n} \left(\frac{1}{R_S}\right)^{(n-1)/n} \quad \text{(S3)}$$

$$C = Y_0^{1/n} \left(\frac{1}{R_S} + \frac{1}{R_f}\right)^{(n-1)/n} \quad \text{(S4)}$$

SB. Carbonization parameters

Table S1. Carbonization time and temperature for prawn and squid chitin with associated S_{BET}, capacitance and % retention.

**Carbon precursor**	**Temperature**	**Carbonization time**	**S_{BET} ($m^2\ g^{-1}$)**	**C ($F\ g^{-1}$) 1st cycle**	**% retention After 1000th cycle**
Prawn chitin	1000 °C	10 min	9.74	3	65.3
		30 min	22.56	4	70.5
		**1 h**	**84.96**	**15**	**92.1**
		2 h	81.24	14	90.4
		3 h	80.91	11	90.8
		4 h	79.22	12	91.1
	900 °C	1 h	65.11	11	75.1
	500 °C		25.64	5	45.4
Squid chitin	1000 °C	10 min	32.2	1	88.1
		30 min	86.2	8	95.4
		**1 h**	**149.3**	**20**	**95.7**
		2 h	131.4	18	95.0
		3 h	124.1	18	95.0
		4 h	123.4	16	93.6
	900 °C	1 h	120.9	15	93.4
	500 °C		76.2	8	92.5

SC. Figures

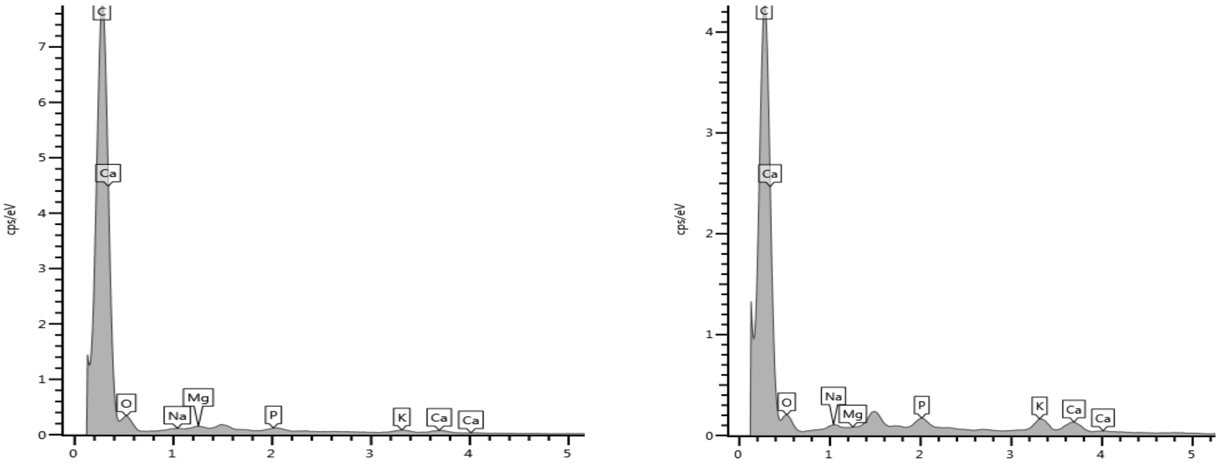

(a) (b)

Figure S1. EDX analysis of squid (**a**) and prawn (**b**) chitin-based carbons

Squid chitin–based carbon

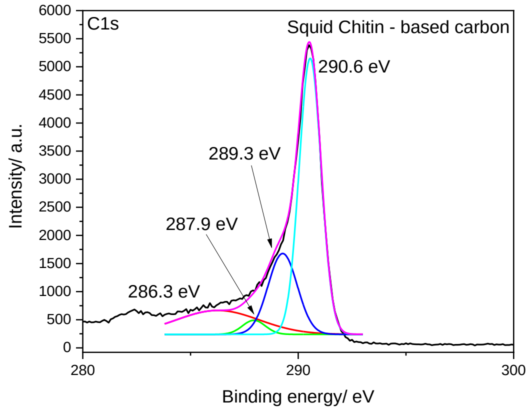

(a)

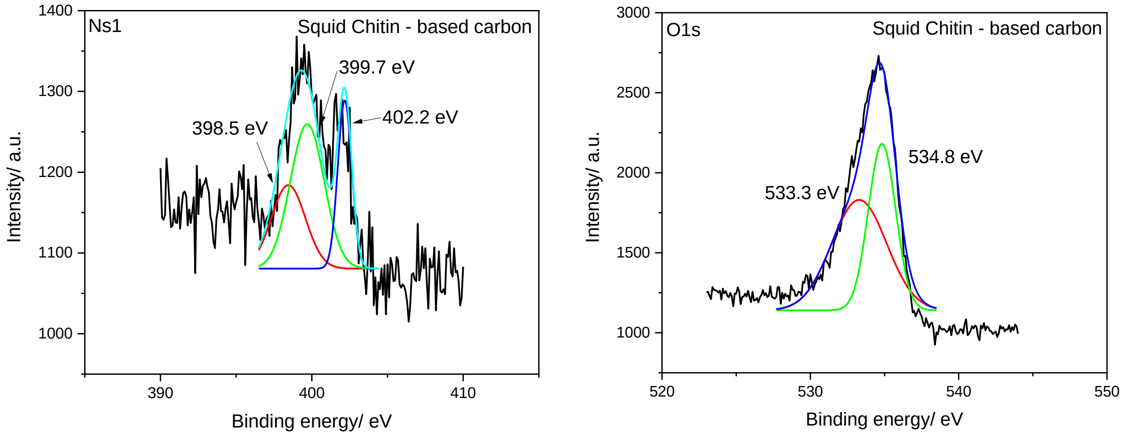

(b) (c)

Figure S2. Deconvolution of the peaks of the XPS survey spectra for squid chitin-based carbon carbonized for 1 h at 1000 ºC: C1s (**a**), O1s (**b**) and N1s (**c**).

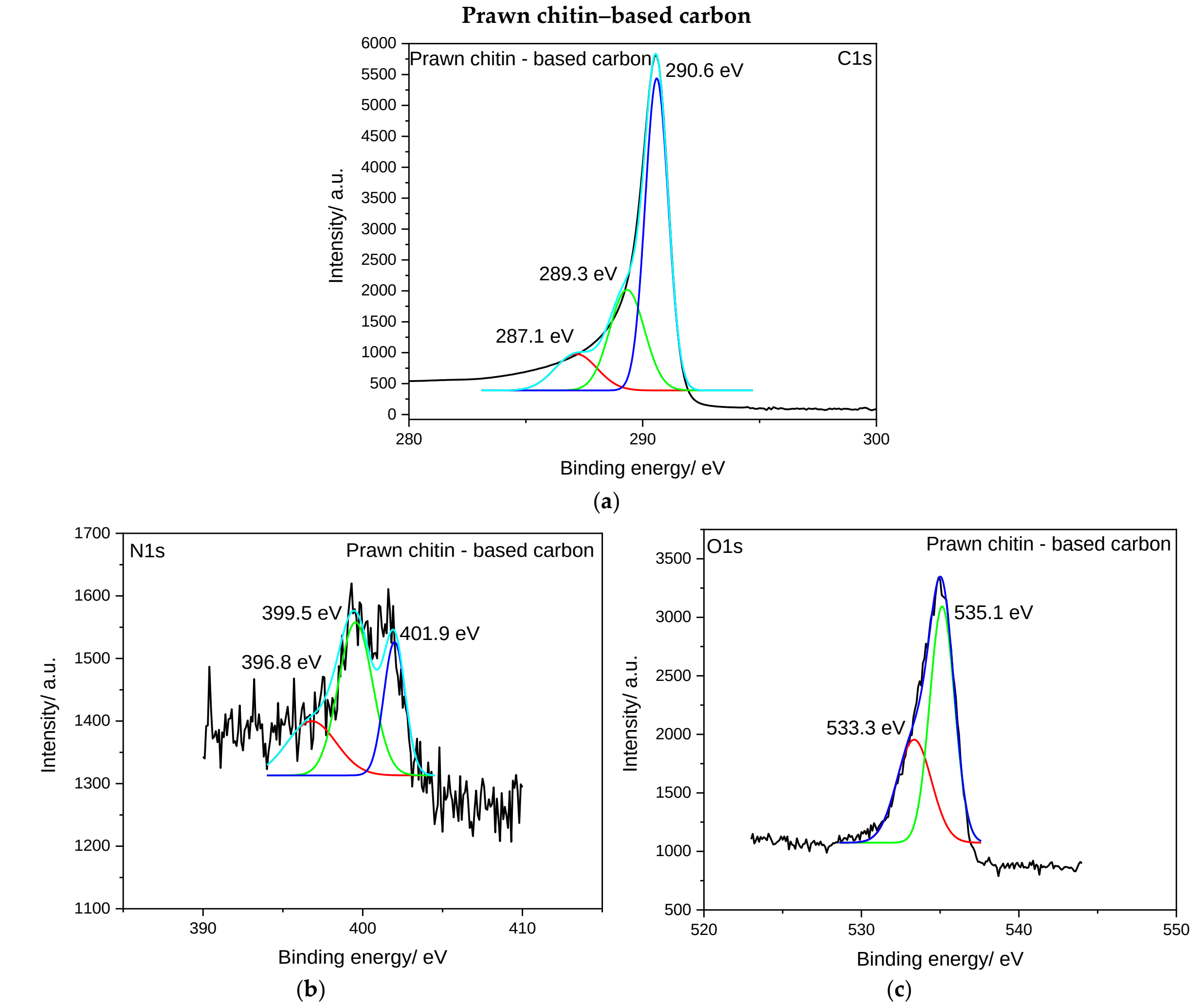

Figure S3. Deconvolution of the peaks of the XPS survey spectra for prawn chitin-based carbon carbonized for 1 h at 1000 ºC: C1s (**a**), O1s (**b**) and N1s (**c**).

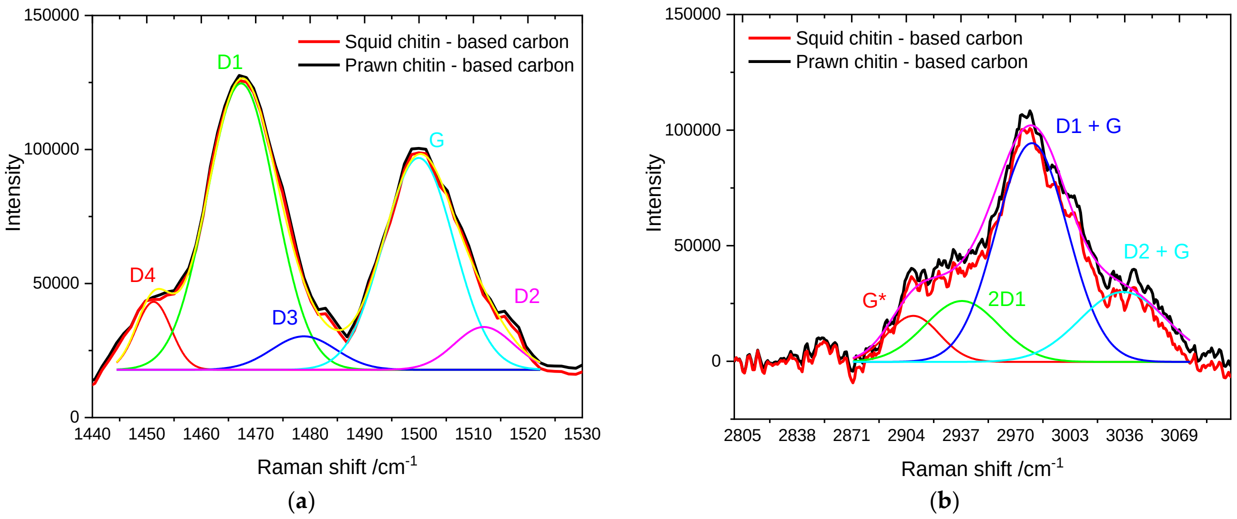

Figure S4. 1st Raman region (**a**) and 2nd Raman region (**b**) of the Raman spectra of the chitin-based carbons.

SD. Elements of the equivalent circuits for the EIS analysis

Table S2. Parameters of all elements of the equivalent circuit (RRQ) for prawn chitin-based carbon.

**Prawn Chitin-based carbon (RRQ)**		$C = Y_0^{1/n}\left(\frac{1}{R_S}+\frac{1}{R_f}\right)^{(n-1)/n}$		
**E/V**	**R_1**	**Y_0**	**n**	**R_2**
0	1062.137	9.40×10^{-6}	0.753294	97,779.67
0.1	154.6942	7.01×10^{-6}	0.801311	84,951.24
0.2	156.3942	6.25×10^{-6}	0.813279	104,855.3
0.3	158.1644	5.49×10^{-6}	0.827178	130,996.8
0.4	160.286	4.76×10^{-6}	0.842533	172,380.4
0.5	161.8159	4.28×10^{-6}	0.854853	244,247.6
0.6	162.7571	4.08×10^{-6}	0.861294	329,510.9
0.7	162.8594	4.15×10^{-6}	0.862457	373,946.4
0.8	162.5991	4.38×10^{-6}	0.860476	335,378.6
0.9	162.0957	4.75×10^{-6}	0.855922	264,149.4
1	161.3368	5.26×10^{-6}	0.848523	185,343.2

Table S3. Parameters of all elements of the equivalent circuit (RQ) for squid chitin-based carbon

**Squid Chitin (RQ)**		$\mathbf{C = Y_0^{1/n}\left(\frac{1}{R_S}\right)^{(n-1)/n}}$	
**E/V**	**R_1**	**Y_0**	**n**
0	135.368	4.46×10^{-5}	0.79585
0.1	134.5941	4.21×10^{-5}	0.791452
0.2	133.7856	4.26×10^{-5}	0.785258
0.3	133.1522	4.36×10^{-5}	0.77885
0.4	132.8227	4.33×10^{-5}	0.775744
0.5	132.3068	4.33×10^{-5}	0.774201
0.6	131.6339	4.44×10^{-5}	0.771833
0.7	130.6132	4.79×10^{-5}	0.763963
0.8	129.5429	5.41×10^{-5}	0.751643
0.9	128.3261	6.33×10^{-5}	0.733949
1	127.0343	7.63×10^{-5}	0.709736

SE. Preliminary results – Electrochemical Studies

The study of how the electrolyte can affect the electrochemical behaviour of the half-cell set-up was performed. Two aqueous electrolytes were chosen for this study 1 mol L^{-1} H_2SO_4 and 1 mol L^{-1} KOH. Since the squid chitin-based carbon presented the most promising results, it was the chosen material for this work.

The characterization techniques are presented in the section Materials and Methods. The results are discussed in the main text.

Table S4. Specific capacitance and % retention after 1000 and 5000 cycles of squid chitin-based carbons sample (1 A g^{-1}) in DES, 1 mol L^{-1} H_2SO_4 and 1 mol L^{-1} KOH.

	Electrochemistry 30 °C		
**Squid Chitin 1000 °C 1h**	**C (F g^{-1}) 1st cycle**	**% retention After 1000th cycle**	**% retention After 5000th cycle**
DES	20 ± 1	96	93
1 mol L^{-1} H_2SO_4	23 ± 2	86	45
1 mol L^{-1} KOH	12 ± 3	80	33

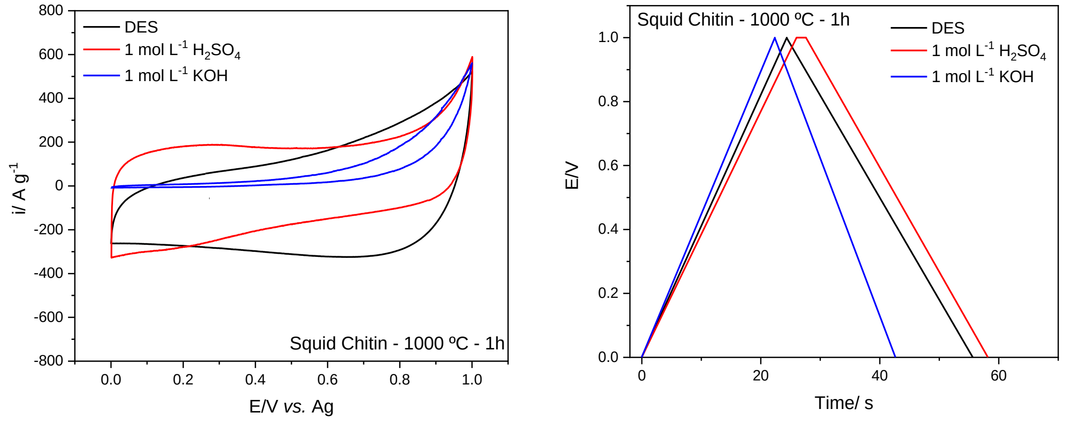

(**a**) (**b**)

Figure S5. Electrochemical study of the squid chitin-based carbon, in DES, 1 mol L^{-1} H_2SO_4 and 1 mol L^{-1} KOH (**a**) cyclic voltammetry at 50 mV s^{-1}, and (**b**) galvanostatic charge-discharge curves recorded with current density 1 A.g^{-1}, at 30 °C.

SF. References

36. Brandão, A.T.S.C.; Costa, R.; Silva, A.F.; Pereira, C.M. Sustainable Preparation of Nanoporous Carbons via Dry Ball Milling: Electrochemical Studies Using Nanocarbon Composite Electrodes and a Deep Eutectic Solvent as Electrolyte. *Nanomaterials* **2021**, *11*, 3258.

38. Brandão, A.T.S.C.; Rosoiu, S.; Costa, R.; Lazar, O.A.; Silva, A.F.; Anicai, L.; Pereira, C.M.; Enachescu, M. Characterization and electrochemical studies of MWCNTs decorated with Ag nanoparticles through pulse reversed current electrodeposition using a deep eutectic solvent for energy storage applications. *J. Mater. Res. Technol.* **2021**, *15*, 342–359.

41. Pimenta, M.A.; Dresselhaus, G.; Dresselhaus, M.S.; Cançado, L.G.; Jorio, A.; Saito, R. Studying disorder in graphite-based systems by Raman spectroscopy. *Phys. Chem. Chem. Phys.* **2007**, *9*, 1276–1290.

42. Salomé, S.; Pereira, N.M.; Ferreira, E.S.; Pereira, C.M.; Silva, A.F. Tin electrodeposition from choline chloride based solvent: Influence of the hydrogen bond donors. *J. Electroanal. Chem.* **2013**, *703*, 80–87.

43. Stoller, M.D.; Ruoff, R.S. Best practice methods for determining an electrode material's performance for ultracapacitors. *Energy Environ. Sci.* **2010**, *3*, 1294–1301.

69. Figueiredo, M.; Gomes, C.; Costa, R.; Martins, A.; Pereira, C.M.; Silva, F. Differential capacity of a deep eutectic solvent based on choline chloride and glycerol on solid electrodes. *Electrochim. Acta* **2009**, *54*, 2630–2634.
